# Isolation and expression of the human gametocyte-specific factor 1 gene (*GTSF1*) in fetal ovary, oocytes, and preimplantation embryos

**DOI:** 10.1007/s10815-016-0795-0

**Published:** 2016-09-19

**Authors:** John Huntriss, Jianping Lu, Karen Hemmings, Rosemary Bayne, Richard Anderson, Anthony Rutherford, Adam Balen, Kay Elder, Helen M. Picton

**Affiliations:** 1 Division of Reproduction and Early Development, Leeds Institute of Cardiovascular and Metabolic Medicine, Clarendon Way, University of Leeds, Leeds, LS2 9JT UK; 2 MRC Centre for Reproductive Health, University of Edinburgh, Queen’s Medical Research Institute, Edinburgh, EH16 4TJ UK; 3 Leeds Centre for Reproductive Medicine, Leeds Teaching Hospital NHS Trust, Seacroft Hospital, York Road, Leeds, LS14 6UH UK; 4 Bourn Hall Clinic, Cambridge, CB23 2TN UK

**Keywords:** Oocyte, Ovarian follicle, *GTSF1*, cue110, *FAM112B*, piRNA

## Abstract

**Purpose:**

Gametocyte-specific factor 1 has been shown in other species to be required for the silencing of retrotransposons via the Piwi-interacting RNA (piRNA) pathway. In this study, we aimed to isolate and assess expression of transcripts of the gametocyte-specific factor 1 (*GTSF1*) gene in the human female germline and in preimplantation embryos.

**Methods:**

Complementary DNA (cDNA) libraries from human fetal ovaries and testes, human oocytes and preimplantation embryos and ovarian follicles isolated from an adult ovarian cortex biopsy were used to as templates for PCR, cloning and sequencing, and real time PCR experiments of GTSF1 expression.

**Results:**

*GTSF1* cDNA clones that covered the entire coding region were isolated from human oocytes and preimplantation embryos. *GTSF1* mRNA expression was detected in archived cDNAs from staged human ovarian follicles, germinal vesicle (GV) stage oocytes, metaphase II oocytes, and morula and blastocyst stage preimplantation embryos. Within the adult female germline, expression was highest in GV oocytes. *GTSF1* mRNA expression was also assessed in human fetal ovary and was observed to increase during gestation, from 8 to 21 weeks, during which time oogonia enter meiosis and primordial follicle formation first occurs. In human fetal testis, *GTSF1* expression also increased from 8 to 19 weeks.

**Conclusions:**

To our knowledge, this report is the first to describe the expression of the human *GTSF1* gene in human gametes and preimplantation embryos.

**Electronic supplementary material:**

The online version of this article (doi:10.1007/s10815-016-0795-0) contains supplementary material, which is available to authorized users.

## Introduction

The analysis of genes that are expressed in murine germ cells or whole ovaries has led to the identification of a novel gene, gametocyte-specific factor 1 (*Gtsf1*), also known as the computationally obtained undifferentiated and/or embryonic stem cell-specific 110 gene (Cue110) [1–3]. *Gtsf1* encodes a 167-amino acid protein that is a member of the Uncharacterized Protein Family 0224 (UPF0224).

Three recent reports have revealed that in *Drosophila*, gametocyte-specific factor 1 (DmGTSF1), is essential for P-element-induced wimpy testis (PIWI)-interacting RNA (piRNA)-mediated transcriptional repression and histone H3K9me3-mediated repression of transposons and their neighboring genes in the ovary [4–6]. DmGTSF1 interacts with Piwi via its C-terminal tail [4] and the two CHHC zinc-finger motifs within the DmGTSF1 protein are required for its activity [5]. In the mouse, the *Gtsf1* gene is essential for spermatogenesis and has been revealed to function in transposon suppression in the mouse testes since increased expression of the long interspersed nucleotide element (line 1) and the intracisternal A-particle (IAP) retrotransposons were observed to occur in *Gtsf1*-null male mice [7]. The same study showed that *Gtsf1*-null male mice are sterile due to massive apoptosis of their germ cells and the cessation of meiotic progression before the zygotene stage. In humans, these observations are reflected by the observation that the testes of high infertility risk (HIR) cryptorchidism patients have reduced *GTSF1* expression and corresponding over-expression of LINE1 (L1) retrotransposons [8]. *GTSF1* has also been identified as a bovine spermatozoal transcript [9]. Elsewhere, *GTSF1* transcript expression has been observed to be up-regulated in certain leukemias and in lymphomas [10–14].

The precise role of the *Gtsf1* gene in the mammalian female germline is less clear since *Gtsf1*-null female mice were observed to be fertile [7]. The expression of *Gtsf1* transcripts has been detected in adult mouse primordial and primary follicles, whilst Gtsf1 protein has been detected in germ cell clusters and primordial follicles in newborn ovaries and in the oocytes of all follicle stages [3]. Significantly, *Gtsf1* was identified among the transcripts that were down-regulated in ovaries from mice deficient for the *Nobox* gene [2, 3]. Whilst not essential for fertility, a role for *Gtsf1* is implied during oogenesis and/or early embryonic development since other genes, with diverse functions during oogenesis, are also down-regulated in *Nobox*-deficient mice [15].

To our knowledge, this is the first report to describe cloning and expression analysis of *GTSF1* transcripts in human fetal ovaries and testis, adult human ovarian follicles, GV and metaphase II oocytes, and in preimplantation embryos.

## Materials and methods

### Samples

Human fetal ovaries and testes were obtained with informed consent, according to published methods [16]. Ethical approval was from the Lothian Research Ethics Committee (study code LREC 08/S1101/1). Briefly, human fetal gonads (gestations between 8 and 21 weeks) were obtained following medical termination of pregnancy and were subsequently snap-frozen and stored at −80 °C. RNA was extracted using the Qiagen RNeasy Mini Kit (14 weeks gestation onwards) or Qiagen RNeasy Micro Kit (8–12 weeks gestation) (Qiagen, Crawley, UK) with 500 ng RNA used for first-strand cDNA synthesis using the Superscript Vilo Reverse Transcriptase Master Mix (Life Technologies, Paisley, UK).

Methods for sample preparation of the cDNA libraries from human ovarian follicles and oocytes used in the present study were performed according to published methods [17, 18]. An ovarian cortex biopsy was donated for research by a 22-year-old woman attending Leeds General Infirmary hospital for gynaecological surgery under an ethically approved protocol as described [17]. Following harvest, the tissue was cryopreserved. Briefly, human ovarian follicles were isolated from the frozen-thawed ovarian cortex biopsy by dissection and staged according to size and morphology. Samples were collected from primordial follicles (*n* = 28), primordial/early primary follicles (*n* = 45), and primary follicles (*n* = 7), through to secondary stage follicles (*n* = 7). Diameters used for follicle staging were primordial follicles 34–38 mm, early primary follicles 34–53 mm, primary follicles 52–62 mm, and secondary follicles 62–86 mm. Mature metaphase II (MII) oocytes and preimplantation embryos that were surplus to requirement for clinical treatment were donated for research by patients attending the assisted conception unit at Leeds General Infirmary (Leeds, United Kingdom). Additionally, germinal vesicle (GV)-stage oocytes and cumulus and mural granulosa cells were harvested from non-luteinised antral follicles of 5 mm diameter that were aspirated from two patients during immature oocyte recovery as part of an in vitro maturation program as detailed in the protocol of Wynn et al. 1998 [19]. These oocytes and their surrounding granulosa cells had not been exposed to hCG prior to recovery. Cleavage stage embryos that were surplus to the patients’ treatment needs were donated for research following embryo transfer on day 2, post insemination. Blastocysts were generated in vitro after a further 3–5 days of culture according to the methods detailed by Houghton et al. 2002 and, Ghassemifar et al. 2003 [20, 21]. Additionally, Cryopreserved embryos which were surplus to the patients’ treatment requirements were donated for research under informed consent by couples attending Bourn Hall Clinic (Cambridge, United Kingdom). Cryopreserved embryos were thawed and cultured to the blastocyst stage in the HFEA licensed research laboratories in Leeds according to the methods of Houghton et al. (2002) and, Ghassemifar et al. (2003) [20, 21]. In summary, both fresh and frozen-thawed, cleavage staged preimplantation embryos were individually cultured in 4 μl droplets of Earle’s Balanced Salt Solution, supplemented with 1 mM glucose, 5 mM lactate, 0.47 mM sodium pyruvate, 0.5 % (*v*/*v*) human serum albumin (Zenalb 20; Bio Products Laboratory), and amino acids at close-to-physiological concentrations, as defined by Tay et al. 1997 [22], under embryo tested mineral oil at 37 °C under 5 % CO_2_ in air. At the end of culture, embryos were washed in Ca^2+^ and Mg^2+^ free phosphate buffered saline at 4 °C (Invitrogen, UK), before being snap frozen in liquid nitrogen in 20 μl Dynal lysis buffer (Life Technologies, Ltd). All samples were obtained after informed consent under ethically approved protocols at the LGI, which were licensed in the UK by the Human Fertilisation and Embryology Authority.

### Reverse transcription and cDNA amplification

**Fig. 1:**
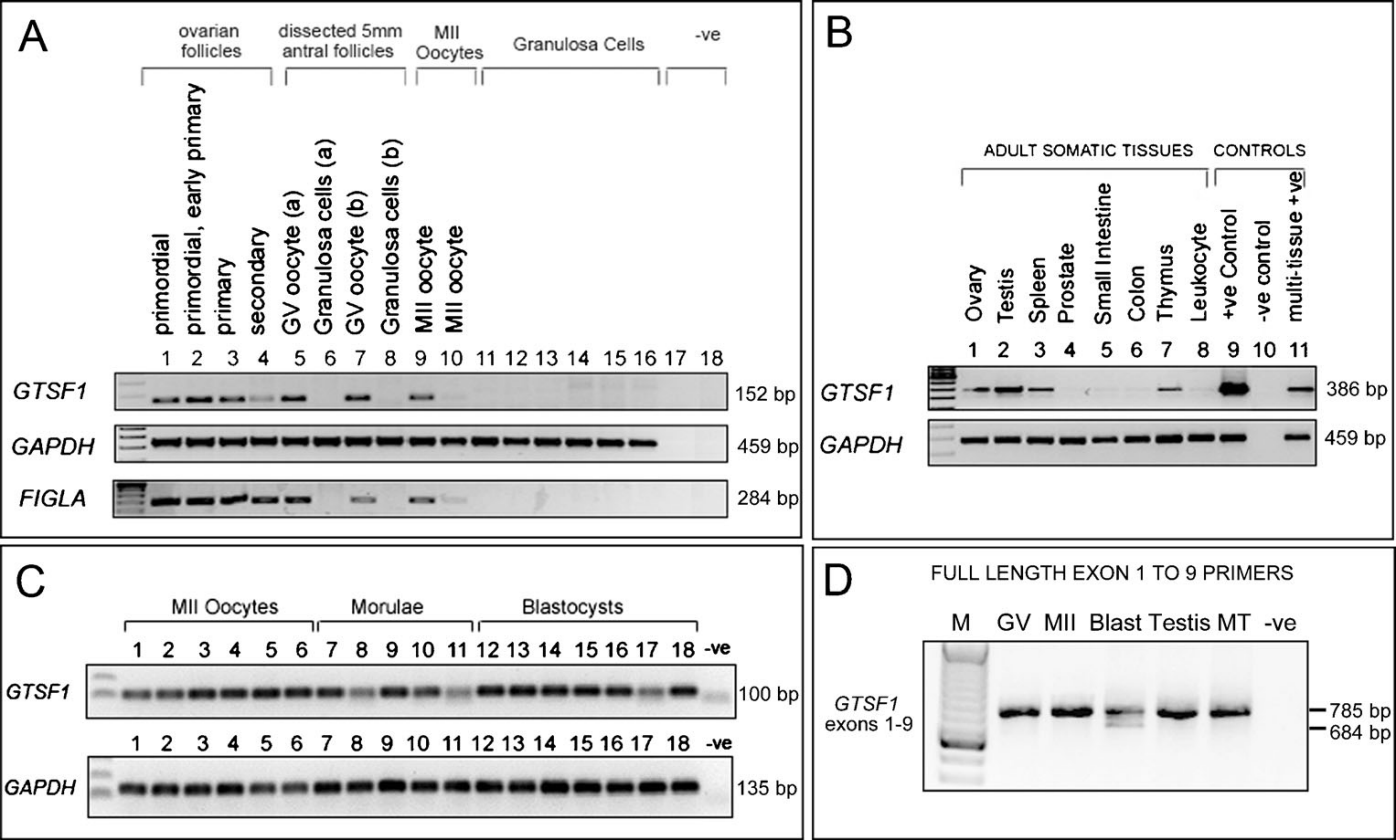
Expression of *GTSF1* transcripts in the human female germline, preimplantation embryos, and adult tissues. **a** Expression of *GTSF1* (primers 7 F and 9R2: Size = 152 base pairs), *GAPDH*, and *FIGLA* gene transcripts by reverse transcription RT-PCR in amplified cDNA samples derived from human ovarian follicles, oocytes, and granulosa cells. The *FIGLA* PCR demonstrates the presence of oocyte cDNA in the ovarian follicle samples. Lane (1) Primordial follicles (*n* = 28 pooled follicles), (2) pooled primordial/early primary follicles (*n* = 45), (3) pooled primary follicles (*n* = 7), and (4) pooled secondary follicles (*n* = 7). Lanes (5) to (8) are cDNAs generated from two dissected 5 mm antral follicles (follicles a and b). Lane (5) Denuded single human GV stage oocytes isolated from the compact cumulus-enclosed oocyte complexes of a 5 mm non-luteinised antral follicle (follicle a), lane (6) granulosa cells isolated from follicle a. Lane (7) Denuded single human GV stage oocytes isolated from the compact cumulus-enclosed oocyte complexes of a 5 mm nonluteinised antral follicle (follicle b), lane 8 granulosa cells isolated from follicle b. Lanes (9) and (10) single MII oocytes, lanes 11 to 16 cDNA from cumulus granulosa cells isolated from 5–10 mm non-luteinised antral follicles containing failed fertilization metaphase II oocytes. Lanes (17) and (18) are negative PCR controls (no template, −ve). *Only weak PCR products for *FIGLA* and *GTSF1* were observed in one of the MII oocytes tested in this particular assay (lane 10). **b** Expression of *GTSF1* (primers 3 F and 7R) and *GAPDH* in cDNAs from adult human tissue (Clontech Multiple Tissue cDNA MTC panels) and controls. Lanes (1) ovary, (2) testis, (3) spleen, (4) prostate, (5) small intestine, (6) colon, (7) thymus, (8) leukocyte, (9) pooled GV oocyte (positive control, +ve), (10) negative control (no template, −ve), and (11) mixed adult human multiple tissue sample. **c** *GTSF1* RT-PCR (primers *GTSF1* real-time F and *GTSF1* real-time R), in individual human oocytes and preimplantation embryos. cDNAs were derived from pooled metaphase II oocytes (*n* = 6, lanes 1 to 6), morulae (*n* = 5, lanes 7–11), blastocysts (*n* = 7, lanes 12–18), and a negative control (−ve, no template). **d** *GTSF1* RT-PCR covering the entire coding region, from exons 1–9, in cDNAs derived from pooled germinal vesicle (GV) oocytes (*n* = 6), metaphase II oocytes (*n* = 7), blastocysts (*n* = 7), testis and mixed adult human multiple tissue samples (MT)

Ovarian follicle samples, single embryos, and oocytes were collected and lysed at 80 °C in Dynal lysis buffer (Life Technologies, Ltd) and cDNA libraries were generated as previously described [23]. For the ovarian follicle samples as used in Fig. 1a, amplified cDNAs were generated from (i) pooled primordial follicles (*n* = 28), (ii) primordial/early primary follicles (*n* = 45), (iii) primary follicles (*n* = 7), and (iv), secondary stage follicles (n = 7).

Messenger RNA was extracted from preimplantation embryos with oligo-dT Dynabeads, and cDNA was generated using an adaptation of existing cDNA amplification protocols, using 1 μg each of primer (primer 1: 5′-aaacgacggccagtgaattgtaatacgactcactatagggcgct_24_-3′ and primer 2: 5′-aagcagtggtatcaacgcagagtacgcggg-3′) and Superscript II RNAseH- Reverse Transcriptase (Life Technologies, Ltd) and associated reagents with incubation for 2 h at 42 °C. The cDNA was amplified by PCR using an additional 1 μg of each primer, 2 μl 50× Advantage 2 Polymerase (BD Clontech), in a thermal cycler for 32 cycles of 95 °C for 45 s, 65 °C for 6 min 45 s in a total volume of 50 μl.

### Isolation of GTSF1 transcripts and expression analysis

Expression analysis for *GTSF1* was performed using 0.3 μl of amplified cDNA in a 12.5 μl reaction volume using BIOTAQ (BIOLINE). *GTSF1* primers (Table 1) were designed using the Primer3 programme (http://primer3.ut.ee/) and derived from human sequences found in GenBank (*GTSF1* AK098819; *GAPDH* AF261085, NM_144594.2). PCR was performed for 32 cycles for 45 s at each step of 94, 60, and 72 °C. The housekeeping gene (glyceraldehyde-3-phosphate dehydrogenase) *GAPDH* was used as a positive control to demonstrate that the cDNA libraries that were generated from each sample were successful; *GAPDH* primers used in Fig. 1a, b were taken from Weisenberger et al. 2002 [24]. The factor in the germline alpha (*FIGLA*) primers was from Huntriss et al. 2002 [17]. Products were run on 1.2 % agarose gels and visualised using ethidium bromide with reference to 100 base pair DNA size markers (Life Technologies, Ltd). Expression of the human *GTSF1* gene was also analysed using a range of normalized cDNA samples derived from various human tissues (Clontech MTC panels, Clontech).

**Table 1:** PCR primers for conventional and real-time PCR

Gene	Primer name	Sequence 5′ to 3′
*GTSF1*	Exon1F	GGAGGAAGGTGACTGTGAGG
	Exon2F	CACTTGGATTCAGCTTCTTC
	Exon3F	GACCCTGAGAAGCTATTGCA
	Exon7F	CCCTGCGAGCAACATAGTTA
	Exon7R	TAACTATGTTGCTCGCAGGG
	Exon9R	CTGTATCAAAGGTTTATTTGGAAGC
	*GTSF1* real-time F	ATTCAGCTTCTTCATTTCCAACA
	*GTSF1* real-time R	CCTGATTTGATGGTTTTTGTCAT
*FIGLA*	*FIGLA* F1*	GATAAAAAATCTCAACCGTGG
	*FIGLA* R1*	CCCTCCTCTTCTTTCTTC
*GAPDH*	*GAPDH* F^§^	ACGGGAAGCTCACTGGCATGGC
	*GAPDH* R^§^	TCTTACTCCTTGGAGGCCATGTAGG
	*GAPDH* real-time F	TTGTCAAGCTCATTTCCTGGTAT
	*GAPDH* real-time R	TCTCTCTTCCTCTTGTGCTCTTG

*FIGLA primers are from Huntriss et al. [17]

^§^ *GAPDH* primers are from Weisenberger et al. [24]

### Sequencing

*GTSF1* PCR products that were obtained using various primer combinations were cloned into TOPO TA (Life Technologies). Primer sequences are given in Table 1. The M13 primer-amplified PCR products were sequenced at the Biomolecular Analysis Facility, University of Leeds. Sequences of PCR products were obtained in both directions and were identified by the Basic Local Alignment Search Tool (BLAST) http://www.ncbi.nlm.nih.gov/BLAST.

## Real-time PCR

The real-time PCR reaction data was collected using an ABI PRISM 7900HT Real-Time PCR system using the SYBR Green method (Applied Biosystems). PCR primer sequences are described in Table 1. The 25 μl reaction mix contained 2 ng cDNA, 10 pmol of each primer. The PCR protocol was denaturation (95 °C for 10 min), amplification (94 °C 10 s, 60 °C 30 s, and 72 °C 30 s for 45 cycles). For quantitative assessment of *GTSF1* expression in the female germline, real-time PCR was performed on cDNA derived from primordial follicles, primordial/early primary follicles, primary follicles, secondary follicles, pooled cDNA from germinal vesicle (GV) stage oocytes (*n* = 8), pooled metaphase II oocytes (*n* = 8), and commercially available ovary and testis RNA (FirstChoice RNA, Ambion). Each sample was assessed in triplicate. Data are expressed as a percentage of the housekeeping gene *GAPDH* using the formula 2^−ΔCT^ × 100, in which CT is the threshold cycle number. For real-time PCR analysis of *GTSF1* expression in human fetal ovary and testis experiments were performed as described previously [25], with the equivalent of approximately 2.5 ng of cDNA used per reaction in 10 μl reaction volumes. Significant changes in expression across gestation were determined by one-way ANOVA with Tukey’s Multiple Comparison Test (GraphPadPrism 5.0 software). Due to the limiting amounts of cDNA that were available for ovarian follicle samples and human fetal gonads, we were unfortunately unable to assess further housekeeping genes across this series of samples as controls.

## Results

### Isolation and expression of GTSF1

A partial sequence of the *GTSF1* gene was originally isolated during differential display analysis from cDNAs derived from staged human ovarian follicles (J Huntriss, D Miller, HM Picton unpublished data). BLAST searches with this sequence, that were performed prior to the release of the *GSTF1* gene sequence (for any species) in any gene database, initially identified a 100 % match over 138 nucleotides of readable sequence to the human sequences AK057504 and AK098819, both testis-derived transcripts. On translation, the partial sequence exhibited similarity to the Xenopus D7 oocyte protein that is represented by P13007 and NP_001081517 protein sequences. The human sequence was chosen for further study by virtue of the fact that it likely represented a novel human oocyte transcript with sequence similarity to the Xenopus D7 protein gene. Analysis by reverse transcription (RT)-PCR with gene-specific primers encompassing the partial sequence from AK057504 in samples derived from the adult human female germline indicated a pattern of expression consistent with being derived from oocytes (Fig. 1a). Figure 1a shows that amplicons were generated in ovarian follicle cDNAs (primordial through to secondary stages), germinal vesicle-stage (GV) oocytes, and metaphase II (MII) oocytes, and therefore in the same samples that we detected transcripts of *FIGLA*, another transcript observed in oocytes [17].

Subsequent BLAST searches identified the partial sequence as a fragment of novel gene, *FAM112B* (family with sequence similarity 112 member B) mapping to chromosome12q13.2. The *FAM112B* gene encodes a member of an uncharacterized protein family containing a conserved protein domain UPF0224. The gene has been more recently assigned the approved name Gametocyte-specific factor 1 and symbol (*GTSF1*), (GeneID: 121355, Ensembl gene ENSG00000170627). We performed further characterisation of the expression of *GTSF1* using primers designed from exons predicted in current *GTSF1* mRNA sequence NM_144594.2 and the related testis-derived mRNA sequences (AK057504 and AK098819) that pre-dated the NM_144594.2 sequence submission. Expression of *GTSF1* was examined across a range of normalized human cDNA samples utilizing primers from exons 3 and 7 of NM_144594.2 (Fig. 1b) and revealed expression in the ovary, testis, spleen thymus, and in the positive controls (cDNA from pooled GV oocytes (lane 9) and mRNA from multiple-tissues (lane 11). Expression of *GTSF1* transcripts was also assessed in a series of individual MII oocytes and in preimplantation embryos (Fig. 1c). *GTSF1* expression was observed in all tested samples: six additional single human MII oocytes, five morulae, and seven blastocysts. A PCR assay covering the entire coding region was designed upon the release of the NM_144594.2 sequence, utilizing primers designed from *GTSF1* exons 1 and 9. This assay amplified the appropriate-sized PCR product in samples of pooled GV oocytes, pooled MII oocytes, pooled blastocysts, testis, and mixed human adult tissues (Fig. 1d). In addition, a second smaller transcript was observed in blastocysts.

### GTSF1 gene and protein sequence

Human *GTSF1* gene cDNA clones that were obtained from human oocytes and blastocysts were sequenced and data is shown in Supplemental Figure 1. This experimentally-defined transcript sequence spans the entire coding region sequence from exon 1 to 9 and the isolated sequence, and its translation, are in full agreement with the sequences NM_144594.2 and NP_653195 and the gene structure as represented by Ensembl GTSF1-001 splice variant that contains 9 exons (Transcript ID: ENST00000305879). The two CHHC zinc-finger domains (TRM13/UPF0224_CHHC_Znf_dom) are identified in Supplemental Figure 1. In blastocysts, the smaller transcript identified in Fig. 1d, that lacks exon 3, most likely represents the GTSF1-004 transcript (Transcript ID: OTTHUMT00000406189), a processed transcript that has no protein product and matches to ESTs (602638807 F1, 602552556 F1) that are derived from mucoepidermoid carcinoma and embryonal carcinoma, respectively. The *GTSF1* cDNA sequences that were isolated from the PCR products shown in Fig. 1d, were subsequently aligned on the browser (Supplemental Figure 2) using the BLAT tool on the UCSC browser (https://genome.ucsc.edu/FAQ/FAQblat.html). Shown are the blastocyst-specific *GTSF1* transcript variant lacking exon 3 (Track 1), the *GTSF1* transcript sequence as isolated from oocytes, blastocysts and all other stages of oogenesis and preimplantation development (Track 2) and the UCSC gene (compiled from RefSeq, GenBank and other sources-Track 3).

## Real-time PCR analysis of *GTSF1* expression in human fetal gonads and the adult female germline

**Fig. 2:**
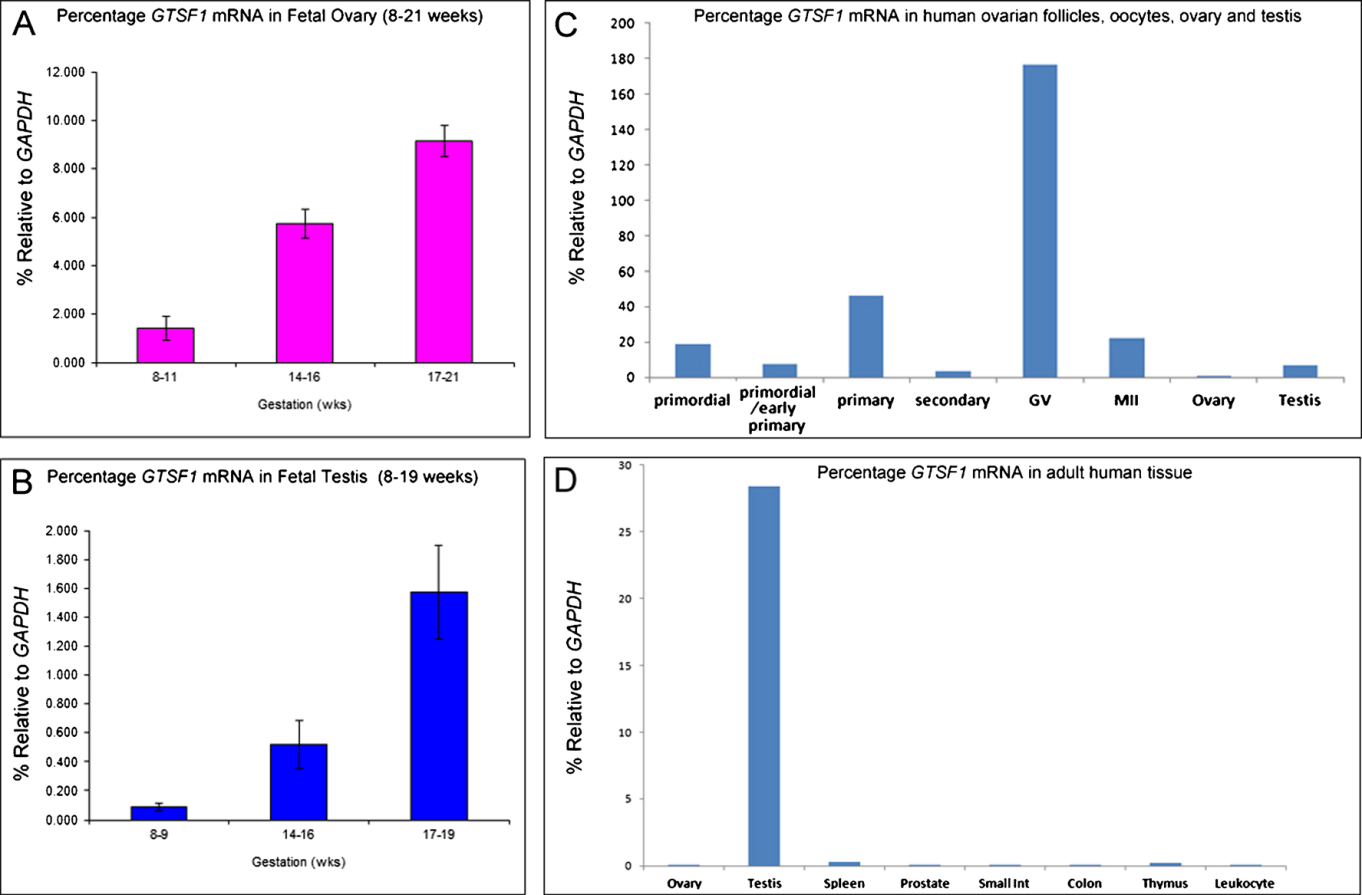
Real-time PCR assessment of *GTSF1* expression. Real-time PCR assessment of *GTSF1* expression is expressed as a percentage of the housekeeping gene *GAPDH* and using primers *GTSF1* real-time F and R and *GAPDH* real-time F and R. **a** *GTSF1* expression in human fetal ovaries at different stages of gestation (total tested range was between 8 and 21 weeks) that were grouped into 8–11 weeks, 14–16 weeks, and 17–21 weeks of gestation, *n* = 4–5 samples per group. *Bars* indicate mean ± sem. **b** *GTSF1* expression in human fetal testis at different stages of gestation (total tested range was between 8 and 19 weeks) that were grouped into 8–9 weeks, 14–16 weeks, and 17–19 weeks of gestation, *n* = 4 samples per group. **c** *GTSF1* expression by real-time PCR in cDNAs from human ovarian follicles (follicle number per sample as described in Fig. 1a) and pooled germinal vesicle (GV) oocytes (*n* = 8), pooled metaphase II oocytes (*n* = 8), and adult gonads. Samples left to right: primordial follicles, primordial/early primary follicles, primary follicles, secondary follicles, pooled cDNA from germinal vesicle (GV) stage oocytes (*n* = 8), pooled metaphase II oocytes (*n* = 8), human ovary (Ambion), and human testis (Ambion). **d** *GTSF1* expression by real-time PCR in cDNAs from human adult human tissue (Clontech Multiple Tissue cDNA panels, tissues as shown in Fig. 1b) and controls

Quantitative measurement of *GTSF1* expression was determined by real-time PCR relative to the housekeeping control gene *GAPDH. GTSF1* expression was assessed in the human fetal ovary and in the human fetal testis at gestations of 8–11 weeks, 14–16 weeks, and 17–21 weeks, using 4–5 samples per group (Fig. 2a). Over these periods, *GTSF1* expression was observed to increase in both the ovary and testis. Significant changes in expression across gestation were determined by one-way ANOVA with Tukey’s Multiple Comparison Test (GraphPadPrism 5.0 software). Thus, ovary fold changes were 8–11 weeks vs. 14–16 weeks: 4-fold increase (*P* < 0.01) and 8–11 week vs. 17–21 weeks, 6.4-fold increase (*P* < 0.001) and 14–16 weeks vs. 17–21 weeks, and 1.6-fold increase (*P* < 0.01). Testis fold changes were 8–9 weeks vs. 14–16 weeks, 5.8-fold increase (not significant), 8–9 weeks vs. 17–19 weeks, 17.4-fold increase (*P* < 0.01) and 14–16 weeks vs. 17–19 weeks, and 3-fold increase (*P* < 0.05). Next, the expression of *GTSF1* during the ovarian follicle growth phase and final stages of oocyte maturation in the adult female ovary was approximated with a series of archived cDNAs [17] from the female germline that included staged ovarian follicles (primordial, primordial/early primary, primary follicles, and secondary follicles) and also included pooled cDNAs derived from GV-staged oocytes and MII oocytes (Fig. 2b). For comparison, cDNAs derived from adult human ovary and testis were included. *GTSF1* expression was detected in all ovarian follicles, and germinal vesicle-stage oocytes and metaphase II oocytes with the highest expression relative to *GAPDH* observed in germinal vesicle-stage oocytes. Finally, real-time PCR was performed using the same range of cDNA samples from adult human tissue as shown in Fig. 1b to reveal that expression of *GTSF1* was highest in the testis (Fig. 2c).

## Conclusions

In this report, we have described the expression of the human *GTSF1* gene from cDNAs derived from fetal gonads, adult human ovarian follicles, GV oocytes, metaphase II oocytes, and preimplantation embryos. The main *GTSF1* transcript isolated from oocytes and preimplantation embryos corresponds to the GTSF1-001 transcript variant of *GTSF1* that encodes a 167 amino acid protein. *GTSF1* is a highly conserved gene that maps to 12q13.2 and has 91 % identity between the mouse and human proteins in agreement with earlier studies [1, 3, 26], with the high conservation indicative of a common function across species.

In cDNAs derived from ovarian follicles that were isolated from the adult ovary, transcripts of *GTSF1* are detected from the primordial follicle stage and are expressed through to the metaphase II oocyte stage. Real-time PCR revealed that highest expression was observed in GV oocytes, with a subsequent decrease in MII oocytes, in agreement with data for mouse *Gtsf1* gene transcripts (http://www.ncbi.nlm.nih.gov/geoprofiles/29792642). Our PCR expression data reveals that *GTSF1* is also expressed during human preimplantation development, being detected in late preimplantation embryos. These observations indicate that expression is activated after zygotic gene activation, and this was supported by our observation of a blastocyst-specific transcript variant of *GTSF1* lacking exon 3. Our expression data for mature oocytes and preimplantation embryos are consistent with that described in the Human Embryo Resource HumER [27]. Our expression data for somatic tissues is consistent with microarray and RNAseq data from other sources that indicate highest expression of the *GTSF1* transcripts within adult tissues occurs in the testis (http://biogps.org/ and http://www.ebi.ac.uk/). Our expression data for oocytes and preimplantation embryos is consistent with microarray data from http://www.ncbi.nlm.nih.gov/geoprofiles (geoprofile IDs 74253766 and 52847566). We were unable to perform analysis of the expression GTSF1 protein in our laboratory; however, GTSF1 protein expression has been described by the Human Protein Atlas ([28]: http://www.proteinatlas.org/ENSG00000170627-GTSF1/tissue). In agreement with our data for the *GTSF1* transcript, this resource reveals that GTSF1 protein (and RNA) expression is largely exclusive to the male and female reproductive tissues. Protein expression is described as being medium in ovarian follicles (but undetectable in ovarian stroma) enriched in the testis, but weakly expressed or undetectable in any other adult human tissues.

Here, we have reported for the first time the elevation of expression of *GTSF1* gene transcripts at the time of entry into meiosis and subsequent primordial follicle formation in the human fetal ovary. This may suggest a particular requirement for *GTSF1* during ovarian programming and early oogenesis and also in GV oocytes in particular. In the male germ line, there is now clear evidence that Piwi-mediated retrotransposon suppression is essential for fertility [29–33], and this includes a requirement for *Gtsf1* in this process [7]. Failure to suppress retrotranposons may affect genomic stability and lead to disease [34–36], and retrotransposons are abundant in germ cells and preimplantation embryos [37–42]. In contrast to the infertility observed in *Gtsf1*-null male mice, *Gtsf1*-null female mice were observed to be fertile with no histological abnormalities in the ovaries [7]. Retrotransposons are known to be silenced in murine primordial ovarian follicles via a piRNA-dependent mechanism, and this is believed to occur in nuage-like structures in the oocytes of primordial follicles [43]. However, mice with nuage-mutant primordial follicles defective for *Mvh*, *Mili*, or *Gasz* remain fertile, despite elevated transposon expression [43]. These studies, which reflect the observations of male-specific sterility vs. female fertility in *Gtsf1*-null mice [7], suggest that the PIWI-pathway in isolation may not be critical for female fertility. Retrotransposon suppression, at least that which is critical for female fertility, may be regulated in the mammalian female germ line by Meiosis arrest female 1 (MARF1) and accordingly, *Marf1* mutant females are infertile [44, 45].

We acknowledge that there are a number of shortcomings in our study. Firstly, for some of the samples, particularly the human ovarian follicles and fetal gonads, we were unable to obtain real-time PCR data for additional housekeeping genes across all stages because of the extremely limiting amounts of cDNA that were available to us. Accordingly, the real-time expression data is only normalised to one housekeeping gene, *GAPDH*. Secondly, we were only able to obtain ovarian follicles from a single patient for this study and therefore, we are not able to confirm whether the *GTSF1* transcript expression patterns observed here are representative of the patterns that would be observed in a wider range of samples from different patients.

Currently, the role of *GTSF1* during mammalian oogenesis remains unclear. The function of *GTSF1*/*Gtsf1* has also been investigated by bioinformatic analysis which has revealed that the two U11-48 K-like CHHC-type zinc finger domains (Pfam: PF05253) within the human GTSF1 protein are found in spliceosomal proteins and tRNA modifying enzymes are predicted to have an RNA binding function [26, 46]. Indeed, the CHHC zinc-finger domain has been observed to specifically bind the 5′ splice site of U12-type introns in spliceosomal U11-48 K proteins [46]. We hypothesize however that although *Gtsf1* appears dispensable for female fertility in mice [7], the retention of the expression of *GTSF1*/*Gtsf1* in the female germline across several species including mouse [1, 3], human (this report), and bovine and ovine oocytes (H.M.Picton, J.Huntriss, J.Lu, G.Liperis- unpublished data, [47, 48]) is indicative of a retained function in the mammalian oocyte and/or early embryo. Of particular interest, the abundance of the GTSF1 protein in murine MII oocytes was observed to be significantly reduced upon postovulatory-ageing [49]; however, further experiments are required to understand the role of this gene in the mammalian oocyte.

## Electronic supplementary material

Below is the link to the electronic supplementary material.

ESM 1 Supplemental figure 1 Experimentally confirmed sequence of the *GTSF1* mRNA isolated from human oocytes. The isolated PCR fragment includes the whole coding sequence (primers Exon1F to Exon 9R). Exon boundaries are indicated by vertical lines. The exon numbering is according to exons identified in sequence NM_144594.2. Arrows indicate the position of *GTSF1* primers Exon1F and Exon9R. The sequence shown is a 100 % match to *GTSF1* from exon 1 to 9. The sequence that lies 5′ of Exon 1 F primer and 3′ of the Exon 9R primer (the *GTSF1* sequence that is not included in the PCR product) is derived from NM_144594.2. The two CHHC Zn-finger motifs are shaded (CHHC in bold). The original readable sequence that was identified during differential display experiments using cDNAs from human ovarian follicles is underlined. In a small number of clones, the first ‘A’ of exon 4 sequenced as ‘G’, although this did not correspond to common single nucleotide polymorphisms, and may have represented an error from PCR amplification. (TIF 778 kb)

ESM 2 Supplemental figure 2 Annotated image from the University of California, Santa Cruz (UCSC) genome browser showing the alignment of the *GTSF1* cDNA sequences that were identified in Fig. 1D. Track 1) The blastocyst-specific *GTSF1* sequence that lacks exon 3; Track 2) The expected near full-length *GTSF1* sequence (exons 1 to 9, covering the entire coding region) that were isolated from oocytes and blastocysts. These *GTSF1* cDNA sequences were aligned on the browser using the BLAT tool on the UCSC browser (https://genome.ucsc.edu/FAQ/FAQblat.html) and are shown with reference to the UCSC gene structure for *GTSF1* (Track 3). (TIF 526 kb)

## References

[1] Yoshimura T, Miyazaki T, Toyoda S, Miyazaki S, Tashiro F, Yamato E (2007). Gene expression pattern of Cue110: a member of the uncharacterized UPF0224 gene family preferentially expressed in germ cells. Gene Expr Patterns.

[2] Choi Y, Qin Y, Berger MF, Ballow DJ, Bulyk ML, Rajkovic A (2007). Microarray analyses of newborn mouse ovaries lacking Nobox. Biol Reprod.

[3] Krotz SP, Ballow DJ, Choi Y, Rajkovic A (2009). Expression and localization of the novel and highly conserved gametocyte-specific factor 1 during oogenesis and spermatogenesis. Fertil Steril.

[4] Donertas D, Sienski G, Brennecke J (2013). Drosophila Gtsf1 is an essential component of the Piwi-mediated transcriptional silencing complex. Genes Dev.

[5] Ohtani H, Iwasaki YW, Shibuya A, Siomi H, Siomi MC, Saito K (2013). DmGTSF1 is necessary for Piwi-piRISC-mediated transcriptional transposon silencing in the Drosophila ovary. Genes Dev.

[6] Muerdter F, Guzzardo PM, Gillis J, Luo Y, Yu Y, Chen C (2013). A genome-wide RNAi screen draws a genetic framework for transposon control and primary piRNA biogenesis in Drosophila. Mol Cell.

[7] Yoshimura T, Toyoda S, Kuramochi-Miyagawa S, Miyazaki T, Miyazaki S, Tashiro F (2009). Gtsf1/Cue110, a gene encoding a protein with two copies of a CHHC Zn-finger motif, is involved in spermatogenesis and retrotransposon suppression in murine testes. Dev Biol.

[8] Hadziselimovic F, Hadziselimovic NO, Demougin P, Krey G, Oakeley E (2015). Piwi-Pathway Alteration Induces LINE-1 Transposon Derepression and Infertility Development in Cryptorchidism. Sex Dev.

[9] Card CJ, Anderson EJ, Zamberlan S, Krieger KE, Kaproth M, Sartini BL (2013). Cryopreserved bovine spermatozoal transcript profile as revealed by high-throughput ribonucleic acid sequencing. Biol Reprod.

[10] Becker H, Marcucci G, Maharry K, Radmacher MD, Mrozek K, Margeson D (2010). Mutations of the Wilms tumor 1 gene (WT1) in older patients with primary cytogenetically normal acute myeloid leukemia: a Cancer and Leukemia Group B study. Blood.

[11] van Kester MS, Borg MK, Zoutman WH, Out-Luiting JJ, Jansen PM, Dreef EJ (2012). A meta-analysis of gene expression data identifies a molecular signature characteristic for tumor-stage mycosis fungoides. J Invest Dermatol.

[12] Boerkamp KM, van der Kooij M, van Steenbeek FG, van Wolferen ME, Groot Koerkamp MJ, van Leenen D (2013). Gene expression profiling of histiocytic sarcomas in a canine model: the predisposed flatcoated retriever dog. PLoS One.

[13] Litvinov IV, Cordeiro B, Huang Y, Zargham H, Pehr K, Dore MA (2014). Ectopic expression of cancer-testis antigens in cutaneous T-cell lymphoma patients. Clin Cancer Res.

[14] Litvinov IV, Netchiporouk E, Cordeiro B, Dore MA, Moreau L, Pehr K (2015). The use of transcriptional profiling to improve personalized diagnosis and management of Cutaneous T-Cell Lymphoma (CTCL). Clin Cancer Res.

[15] Rajkovic A, Pangas SA, Ballow D, Suzumori N, Matzuk MM (2004). NOBOX deficiency disrupts early folliculogenesis and oocyte-specific gene expression. Science.

[16] Bayne RAL, Kinnell HL, Coutts SM, He J, Childs AJ, Anderson RA (2015). GDF9 is transiently expressed in oocytes before follicle formation in the human fetal ovary and is regulated by a novel NOBOX transcript. PLoS One.

[17] Huntriss J, Gosden R, Hinkins M, Oliver B, Miller D, Rutherford AJ (2002). Isolation, characterization and expression of the human Factor In the Germline alpha (FIGLA) gene in ovarian follicles and oocytes. Mol Hum Reprod.

[18] Huntriss J, Hinkins M, Picton HM (2006). cDNA cloning and expression of the human NOBOX gene in oocytes and ovarian follicles. Mol Hum Reprod.

[19] Wynn P, Picton HM, Krapez JA, Rutherford AJ, Balen AH, Gosden RG (1998). Pretreatment with follicle stimulating hormone promotes the numbers of human oocytes reaching metaphase II by in-vitro maturation. Hum Reprod.

[20] Houghton FD, Hawkhead JA, Humpherson PG, Hogg JE, Balen AH, Rutherford AJ (2002). Non-invasive amino acid turnover predicts human embryo developmental capacity. Hum Reprod.

[21] Ghassemifar MR, Eckert JJ, Houghton FD, Picton HM, Leese HJ, Fleming TP (2003). Gene expression regulating epithelial intercellular junction biogenesis during human blastocyst development in vitro. Mol Hum Reprod.

[22] Tay JI, Rutherford AJ, Killick SR, Maguiness SD, Partridge RJ, Leese HJ (1997). Human tubal fluid: production, nutrient composition and response to adrenergic agents. Hum Reprod.

[23] Huntriss JD, Hemmings KE, Hinkins M, Rutherford AJ, Sturmey RG, Elder K (2013). Variable imprinting of the MEST gene in human preimplantation embryos. Eur J Hum Genet.

[24] Weisenberger DJ, Velicescu M, Preciado-Lopez MA, Gonzales FA, Tsai YC, Liang G (2002). Identification and characterization of alternatively spliced variants of DNA methyltransferase 3a in mammalian cells. Gene.

[25] Bayne RA, Eddie SL, Collins CS, Childs AJ, Jabbour HN, Anderson RA (2009). Prostaglandin E2 as a regulator of germ cells during ovarian development. J Clin Endocrinol Metab.

[26] Andreeva A, Tidow H (2008). A novel CHHC Zn-finger domain found in spliceosomal proteins and tRNA modifying enzymes. Bioinformatics.

[27] Vassena R, Boue S, Gonzalez-Roca E, Aran B, Auer H, Veiga A (2011). Waves of early transcriptional activation and pluripotency program initiation during human preimplantation development. Development.

[28] Uhlén M, Fagerberg L, Hallström BM, Lindskog C, Oksvold P, Mardinoglu A (2015). Proteomics. Tissue-based map of the human proteome. Science.

[29] Ma L, Buchold GM, Greenbaum MP, Roy A, Burns KH, Zhu H (2009). GASZ is essential for male meiosis and suppression of retrotransposon expression in the male germline. PLoS Genet.

[30] Shoji M, Tanaka T, Hosokawa M, Reuter M, Stark A, Kato Y (2009). The TDRD9-MIWI2 complex is essential for piRNA-mediated retrotransposon silencing in the mouse male germline. Dev Cell.

[31] Frost RJ, Hamra FK, Richardson JA, Qi X, Bassel-Duby R, Olson EN (2010). MOV10L1 is necessary for protection of spermatocytes against retrotransposons by Piwi-interacting RNAs. Proc Natl Acad Sci U S A.

[32] Reuter M, Berninger P, Chuma S, Shah H, Hosokawa M, Funaya C (2011). Miwi catalysis is required for piRNA amplification-independent LINE1 transposon silencing. Nature.

[33] Pastor WA, Stroud H, Nee K, Liu W, Pezic D, Manakov S (2014). MORC1 represses transposable elements in the mouse male germline. Nat Commun.

[34] Burns KH, Boeke JD (2012). Human transposon tectonics. Cell.

[35] Shukla R, Upton KR, Munoz-Lopez M, Gerhardt DJ, Fisher ME, Nguyen T (2013). Endogenous retrotransposition activates oncogenic pathways in hepatocellular carcinoma. Cell.

[36] Levin HL, Moran JV (2011). Dynamic interactions between transposable elements and their hosts. Nat Rev Genet.

[37] Kano H, Godoy I, Courtney C, Vetter MR, Gerton GL, Ostertag EM (2009). L1 retrotransposition occurs mainly in embryogenesis and creates somatic mosaicism. Genes Dev.

[38] Packer AI, Manova K, Bachvarova RF (1993). A discrete LINE-1 transcript in mouse blastocysts. Dev Biol.

[39] Branciforte D, Martin SL (1994). Developmental and cell type specificity of LINE-1 expression in mouse testis: implications for transposition. Mol Cell Biol.

[40] Ostertag EM, DeBerardinis RJ, Goodier JL, Zhang Y, Yang N, Gerton GL (2002). A mouse model of human L1 retrotransposition. Nat Genet.

[41] Peaston AE, Evsikov AV, Graber JH, de Vries WN, Holbrook AE, Solter D (2004). Retrotransposons regulate host genes in mouse oocytes and preimplantation embryos. Dev Cell.

[42] Georgiou I, Noutsopoulos D, Dimitriadou E, Markopoulos G, Apergi A, Lazaros L (2009). Retrotransposon RNA expression and evidence for retrotransposition events in human oocytes. Hum Mol Genet.

[43] Lim AK, Lorthongpanich C, Chew TG, Tan CW, Shue YT, Balu S (2013). The nuage mediates retrotransposon silencing in mouse primordial ovarian follicles. Development.

[44] Su YQ, Sugiura K, Sun F, Pendola JK, Cox GA, Handel MA (2012). MARF1 regulates essential oogenic processes in mice. Science.

[45] Su YQ, Sun F, Handel MA, Schimenti JC, Eppig JJ (2012). Meiosis arrest female 1 (MARF1) has nuage-like function in mammalian oocytes. Proc Natl Acad Sci U S A.

[46] Tidow H, Andreeva A, Rutherford TJ, Fersht AR (2009). Solution structure of the U11-48K CHHC zinc-finger domain that specifically binds the 5′ splice site of U12-type introns. Structure.

[47] Baillet A, Le Bouffant R, Volff JN, Luangpraseuth A, Poumerol E, Thepot D (2011). TOPAZ1, a novel germ cell-specific expressed gene conserved during evolution across vertebrates. PLoS One.

[48] Liperis G, Iles D, Lu J, Cotterill M, Huntriss J, Picton H. The function of Gametocyte Specific Factor 1 (GTSF1) during ovine oocyte maturation. Society for Reproduction and Fertility 2013. Abstract O027.

[49] Trapphoff T, Heiligentag M, Dankert D, Demond H, Deutsch D, Frohlich T et al. Postovulatory aging affects dynamics of mRNA, expression and localization of maternal effect proteins, spindle integrity and pericentromeric proteins in mouse oocytes. Hum Reprod 2015 (in press). 10.1093/humrep/dev279 PMC5853592 26577303

