# Supplementary figures and images for: Isolation and expression of the human gametocyte-specific factor 1 gene (GTSF1) in fetal ovary, oocytes, and preimplantation embryos
Source: J Assist Reprod Genet. 2016 Sep 19;34(1):23–31. doi: 10.1007/s10815-016-0795-0 (PMC5330970; doi:10.1007/s10815-016-0795-0)

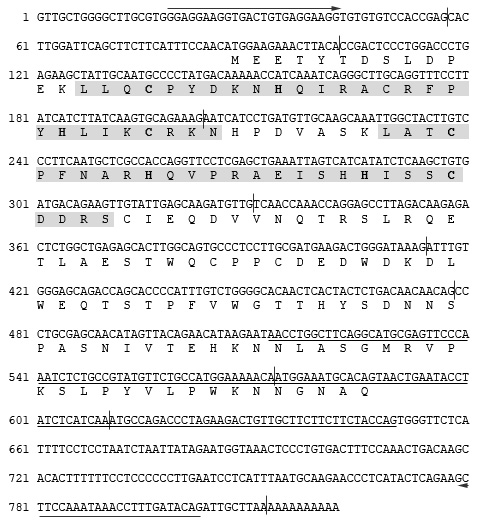

Supplement: Supplementary file 1 — Supplemental figure 1 Experimentally confirmed sequence of the GTSF1 mRNA isolated from human oocytes. The isolated PCR fragment includes the whole coding sequence (primers Exon1F to Exon 9R). Exon boundaries are indicated by vertical lines. The exon numbering is according to exons identified in sequence NM_144594.2. Arrows indicate the position of GTSF1 primers Exon1F and Exon9R. The sequence shown is a 100 % match to GTSF1 from exon 1 to 9. The sequence that lies 5′ of Exon 1 F primer and 3′ of the Exon 9R primer (the GTSF1 sequence that is not included in the PCR product) is derived from NM_144594.2. The two CHHC Zn-finger motifs are shaded (CHHC in bold). The original readable sequence that was identified during differential display experiments using cDNAs from human ovarian follicles is underlined. In a small number of clones, the first ‘A’ of exon 4 sequenced as ‘G’, although this did not correspond to common single nucleotide polymorphisms, and may have represented an error from PCR amplification. (TIF 778 kb) [file 10815_2016_795_MOESM1_ESM.tif]

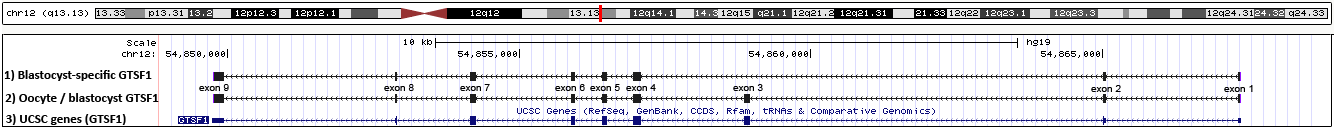

Supplement: Supplementary file 2 — Supplemental figure 2 Annotated image from the University of California, Santa Cruz (UCSC) genome browser showing the alignment of the GTSF1 cDNA sequences that were identified in Fig. 1D. Track 1) The blastocyst-specific GTSF1 sequence that lacks exon 3; Track 2) The expected near full-length GTSF1 sequence (exons 1 to 9, covering the entire coding region) that were isolated from oocytes and blastocysts. These GTSF1 cDNA sequences were aligned on the browser using the BLAT tool on the UCSC browser (https://genome.ucsc.edu/FAQ/FAQblat.html) and are shown with reference to the UCSC gene structure for GTSF1 (Track 3). (TIF 526 kb) [file 10815_2016_795_MOESM2_ESM.tif]
